# Surgical technique for developing a rabbit model of congenital diaphragmatic hernia and tracheal occlusion

**DOI:** 10.1016/j.mex.2019.03.001

**Published:** 2019-03-22

**Authors:** M. Mudri, S.A. Smith, C. Vander Tuin, J. Davidson, T.R.H. Regnault, A. Bütter

**Affiliations:** a Division of General Surgery, Schulich School of Medicine and Dentistry, Western University, London, ON, Canada; b Departments of Obstetrics & Gynaecology and Physiology & Pharmacology, Schulich School of Medicine, Western University, London, ON, Canada; c Division of Paediatric General Surgery, Children’s Hospital London Health Science Centre, Schulich School of Medicine, Western University, London, ON, Canada

**Keywords:** Rabbit model of congenital diaphragmatic hernia and tracheal occlusion, Congenital diaphragmatic hernia (CDH), Tracheal occlusion (TO), Animal model, Rabbit, Fetal surgery

## Abstract

The surgical model of congenital diaphragmatic hernia (CDH) has been utilized in exploring treatments and innovative therapies, such as tracheal occlusion (TO). The rabbit is an excellent surgical model compared to others due to lower cost, ease of care, short gestational period, and large litter size. This model is also ideal in studying lung hypoplasia of CDH because rabbit lung development is most similar to humans as alveolarization begins prior to birth and continues post-natally. However, the surgical technique in creating a rabbit model of CDH is quite difficult and information is lacking on how to establish this model. Therefore, the aim of this paper is to describe:

• Surgical technique in establishing a rabbit model of CDH and TO
• Perioperative care for pregnant rabbit does

## Graphical abstract

**Figure:**
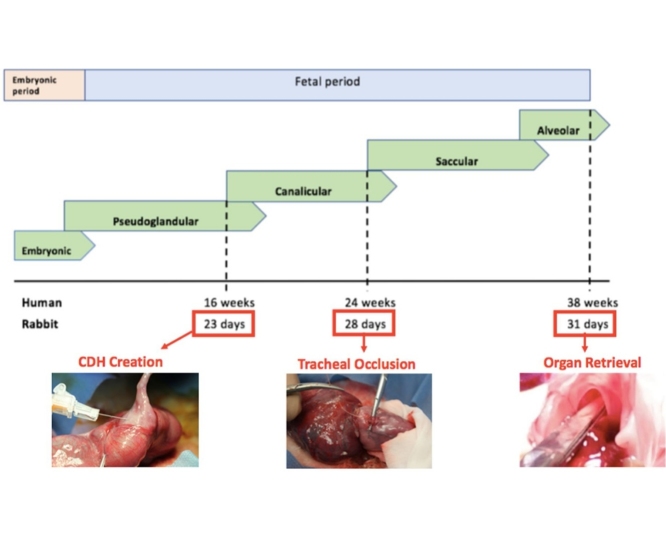

**Specifications Table**

**Table:** 

**Subject Area:**	Medicine and Dentistry
**More specific subject area:**	Congenital diaphragmatic hernia
**Method name:**	Rabbit model of congenital diaphragmatic hernia and tracheal occlusion
**Name and reference of original method:**	Vuckovic A, Herber-Jonat S, Flemmer AW, Roubliova XI, Jani JC. Alveolarization Genes Modulated by Fetal Tracheal Occlusion in the Rabbit Model for Congenital Diaphragmatic Hernia: A Randomized Study. PLoS One 2013;8. doi:10.1371/journal.pone.0069210.
**Resource availability:**	NA

## Method details

Congenital diaphragmatic hernia (CDH) is a birth defect that occurs in every 2000–4000 live births [1,2]. Failure of normal diaphragm development results in a diaphragmatic defect that allows intra-abdominal contents to herniate into the chest cavity [3]. CDH results in lung hypoplasia and pulmonary hypertension, leading to significant morbidity and 20% mortality rate in this population of neonates [1,4]. Despite medical advances is post-natal management of CDH babies, the mortality rate has remained stagnant [1,4]. Therefore, there is a need for fetal treatment that will stimulate lung growth and minimize lung hypoplasia in CDH fetuses to lower the risk of respiratory compromise [1]. The concept of fetal tracheal occlusion (TO) came from the observation that fetuses with laryngeal atresia were born with hyperplastic lungs [1]. TO was first applied in the context of CDH in the early 1990s in the lamb CDH model, where significant lung growth and reversal of pulmonary hypoplasia was noted following TO [1,5,6].

Currently, three types of CDH animal models exist – genetic, pharmacologic, and surgical. Genetic models of CDH are primarily in rodent models with knockout genes [4,7]. This model is perhaps the least popular because the majority of CDH cases in humans are not associated with genetic defects and so the genetic model of CDH is the least applicable to the human condition [4,7].

The pharmacologic model involves the use of a teratogenic herbicide called nitrofen in pregnant rodents which causes CDH and lung hypoplasia in their offspring. This model was first explored in the 1980s by Iritani [3,8]. Typically, 100 mg of nitrofen is dissolved in 1 mL olive oil and administered on day 9 of gestation to induce CDH in rodents [9]. Nitrofen administration induces a right or left-sided diaphragmatic defect in 70–80% of fetuses and causes lung hypoplasia in 100% of fetuses [3,7,10,11]. Advantages of the nitrofen-induced CDH rodent model include inexpensive cost and ease of use [3,7]. However, although nitrofen induces a diaphragmatic defect during early stages of lung development similar to humans, the use of a teratogen to induce this defect is concerning since there is no known teratogen associated CDH in humans [3].

The surgical model of CDH is useful for exploring treatments and innovative therapies in CDH [3,7]. This model has primarily been utilized in sheep and rabbits [3,7]. The first surgical model of CDH was developed using fetal lambs by Delorimier in the 1960s [3,7,12]. The diaphragmatic defect was created surgically at 72–75 days of gestation, which corresponds with the canalicular phase of lung development, with term being 145–149 days of gestation. In the early 1990s, the surgical model of CDH was developed in fetal rabbits by Fauza et al. [13]. There were many advantages identified in using rabbits instead of sheep – lower cost, shorter gestational period of 32 days, and similar lung physiology to humans. In the rabbit model, the diaphragmatic defect is created on gestational day 23, which is also during the pseudoglandular phase of lung development [7]. The lung physiology is more similar to humans compared to other models because in rabbits alveolarization begins prior to birth and completes in the post-natal period, whereas in sheep, alveolarization is nearly complete at birth, and in rodents alveolarization begins in the post-natal period [14]. The feasibility of using rabbits as a surgical model along with their similar lung physiology has made this the most ideal and promising animal model to use for CDH research.

## Perioperative care

**Fig. 1:**
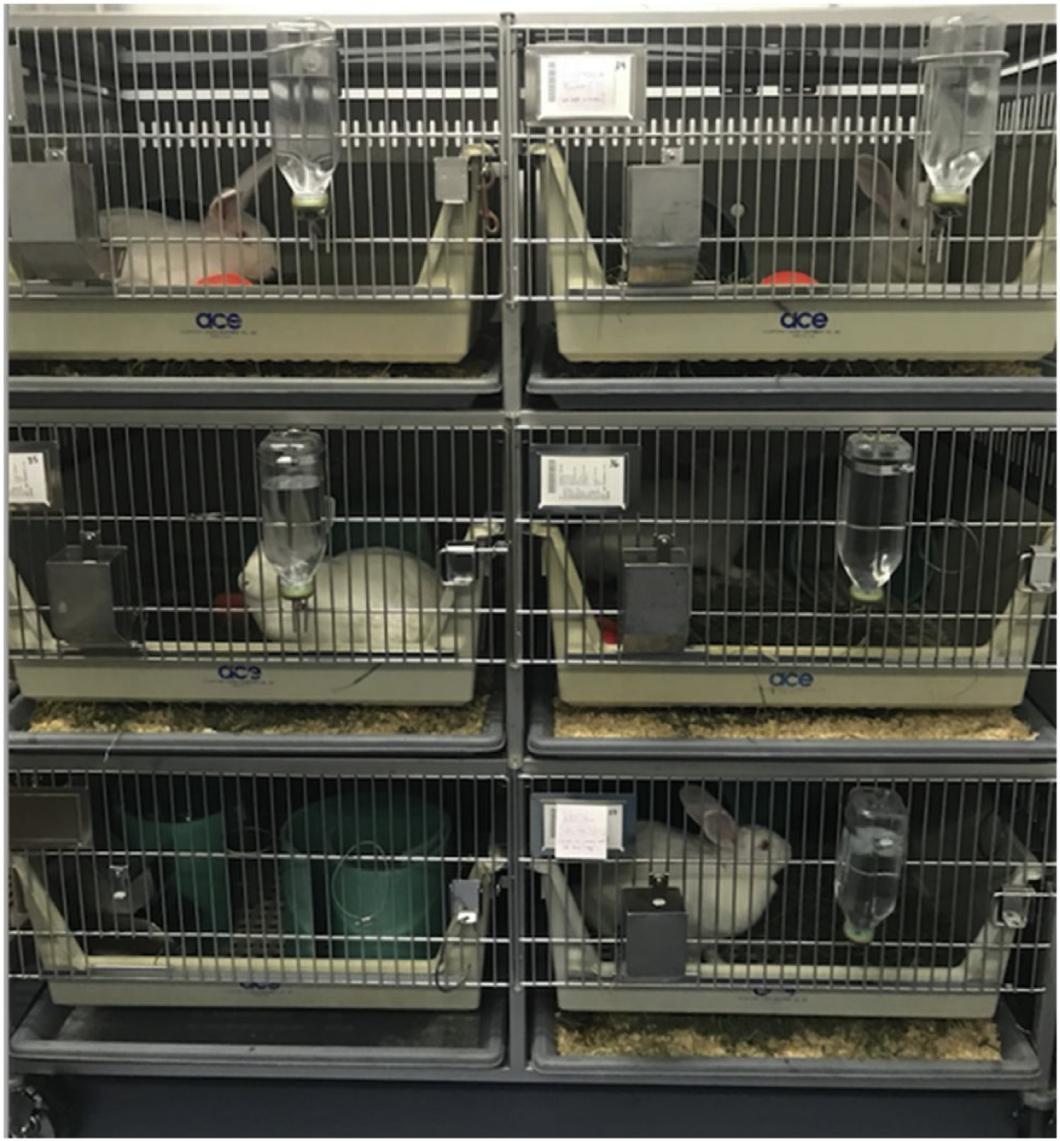
**Housing for Pregnant New Zealand White Rabbits.** Groups of five time-dated New Zealand White rabbits were received at 13 days gestation and housed individually with free access to food, water, and environmental enrichment.

All animal procedures were carried out in strict accordance with the guidelines of the Canadian Council of Animal Care (CCAC) as approved by the Western University Animal Care Committee (AUP 2016-041). All efforts were made to minimize suffering as per the approved protocol and with veterinary oversight. Time dated pregnant New Zealand White rabbits (Charles River, Sherbrooke) arrived at 13 days gestation. Each month, a group of 5 does arrived at our facility and were individually housed with free access to water, food, and environmental enrichment (Fig. 1). Each doe underwent two surgeries, a CDH creation at 23 days gestation and a second surgery, the TO surgery 5 days later at 28 days gestation as previously described [15].

Pre-operatively, each doe received 0.12 mg/kg Buprenorphine SR SQ (conc. 3 mg/mL, Sigma-Aldrich Corporation, St. Louis, MO, USA) for analgesia, Penicillin G 300 000 IU IM (Pfizer Inc., New York, NY, USA) for the prevention of infection, and Depo-Provera 4.5 mg IM (Pfizer Inc.) to reduce the risk of spontaneous abortion. Anesthesia was induced with 5 mg/kg IM Ketamine (conc. 100 mg/mL), 0.15 mg/kg Dexmedetomidine IM (conc. 1 mg/mL, Clearsynth Labs Pvt. Ltd., Mumbai, India), 0.01 mg/kg Glycopyrrolate SQ (conc. 0.2 mg/mL, Omega Laboratories Ltd., Montréal, QC, Canada), and maintained with 5% Isoflurane (Baxter Healthcare Corporation, Deerfield, IL, USA) via facemask. Each doe received a 4 cc/kg bolus of 0.9% NaCl SQ, followed by a maintenance rate of 5 mL/kg/hr. Lacrilube was applied to the eyes and a water circulating pad was used to maintain normothermia intra-operatively. Soft restraints were used on all 4 limbs to secure the doe. Vital signs were monitored and maintained within normal limits including heart rate 180–250, respiratory rate 30–60, and temperature 38–40 °C.

Post-operatively, the does were placed under a red warming light and transferred to their housing cage once mobile. Does were closely monitored with daily weights, intake/output assessment, and incision checks. Meloxicam 0.2 mg/kg SQ (conc. 5 mg/mL, Boehringer Ingelheim Vetmedica Inc., St. Joseph, MO, USA) was administered every 24 h for 2 days for post-operative analgesia.

## CDH creation

**Fig. 2:**
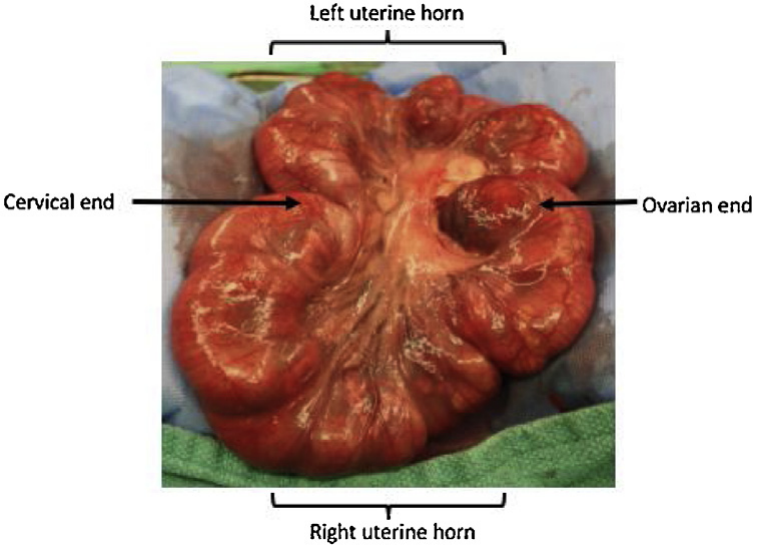
**Bicornuate Rabbit Uterus.** The pregnant New Zealand rabbit uterus is bicornuate with a right and left uterine horn containing multiple fetuses. Fetuses located at the ovarian end were used for surgical experimentation.

**Fig. 3:**
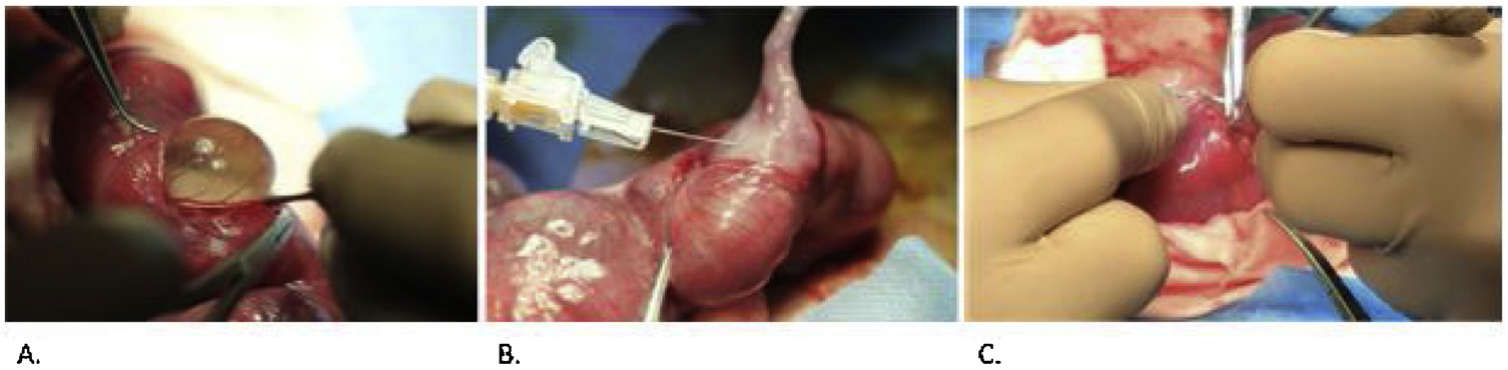
**CDH Creation in the Rabbit Fetus.** A: Hysterotomy with bulging membranes. B: The fetus is minimally exposed with the left upper limb retracted cephalad and a 25-gauge needle is used to make a skin incision at the thoracotomy site. C: The diaphragmatic defect is created by grasping a piece of diaphragm and cutting it off with fine scissors.

CDH creation was performed at 23 days of gestation in a total of 75 fetuses. The does were pre-medicated as described above. The abdominal fur was trimmed, and the abdomen was prepared with 2% chlorhexidine (Laboratoire Atlas Inc., Montréal, QC, Canada) and draped. A lower midline laparotomy incision was made and the uterus was exposed. The number of fetuses in each uterine horn was counted. Two fetuses were chosen from the most ovarian end of each uterine horn, for a total of 4 fetuses per doe (Fig. 2). Fetal position was determined by gentle palpation. A 1 cm hysterotomy was made on the anti-mesometrial side of the uterus with a #15-blade scalpel (Aspen Surgical Products Inc., Caledonia, MI, USA) (Fig. 3). A purse string suture was placed using a 6-0 Prolene® (Ethicon Inc., Somerville, NJ, USA). The left upper limb of the fetus was identified, exposed and retracted cephalad. A left-sided thoracotomy was made at the landmark between the lateral thoracic vessels using a 25-gauge needle (Becton, Dickinson and Company, Franklin Lakes, NJ, USA) and mosquito forceps (Fig. 3). The lung was retracted, exposing the fetal diaphragm. The diaphragm was grasped with mosquito forceps and a piece was cut with fine scissors, creating the diaphragmatic defect (Fig. 3). The chest wall was sutured with a 6-0 Prolene® (Ethicon Inc., Somerville, NJ, USA). The fetus was repositioned into the uterus and the purse string was tied, thus closing the hysterotomy. The laparotomy incision was closed with a 3-0 Vicryl® (Ethicon Inc., Somerville, NJ, USA) in a running fashion. The skin was closed in a running subcuticular fashion with a 4-0 Monocryl® (Ethicon Inc., Somerville, NJ, USA).

In the last set of 5 does, 10 sham CDH fetuses were generated. Surgical protocol was followed as previously described. Following CDH creation, two additional fetuses were chosen at the most ovarian end. A sham CDH was created by performing a left-sided thoracotomy without the creation of a diaphragmatic defect. We recommend that a maximum of 4 fetuses per doe undergo surgery for improved survival of fetuses. We found that adding sham fetuses and increasing the number of operative fetuses to 6 increased the operative time and surgical stress to the doe and fetuses, and resulted in a higher mortality rate.

## Tracheal occlusion

**Fig. 4:**
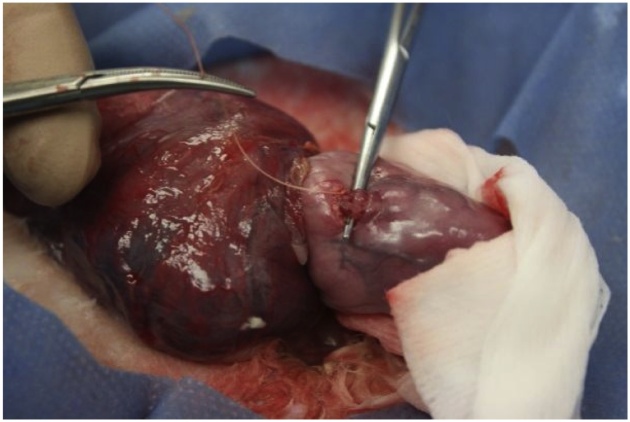
**Tracheal Occlusion in the Rabbit Fetus.** A hysterotomy has been made, the head exposed and neck extended. A small neck incision was used to isolate the trachea which was double ligated with sutures in order to perform the TO.

TO was performed at 28 days gestation in a total of 17 fetuses. The does were pre-medicated as described above. Once anesthesia was induced, the abdomen was prepped and draped in a sterile fashion. The lower laparotomy incision was opened and fetuses were counted. Viability was noted. Half of the potentially viable CDH fetuses were chosen for TO. A hysterotomy was made and the fetal head was exposed. The snout was immediately covered with a saline soaked non-woven sponge to ensure no fetal breathing. A horizontal incision was made at the superior border of the thyroid gland. A fine snap was used to bluntly dissect down to the trachea. Once isolated, the trachea was double ligated with a 4-0 Vicryl® suture (Ethicon Inc., Somerville, NJ, USA) (Fig. 4). Fetal skin was sutured with 6-0 Prolene® (Ethicon Inc., Somerville, NJ, USA). The hysterotomy was closed with 6-0 Prolene® (Ethicon Inc., Somerville, NJ, USA) in a running locking fashion. The doe’s fascia was sutured with 3-0 Vicryl® (Ethicon Inc., Somerville, NJ, USA) and the skin was sutured with 4-0 Monocryl® (Ethicon Inc., Somerville, NJ, USA). Post-operative treatment was as described above.

## Euthanasia and organ retrieval

**Fig. 5:**
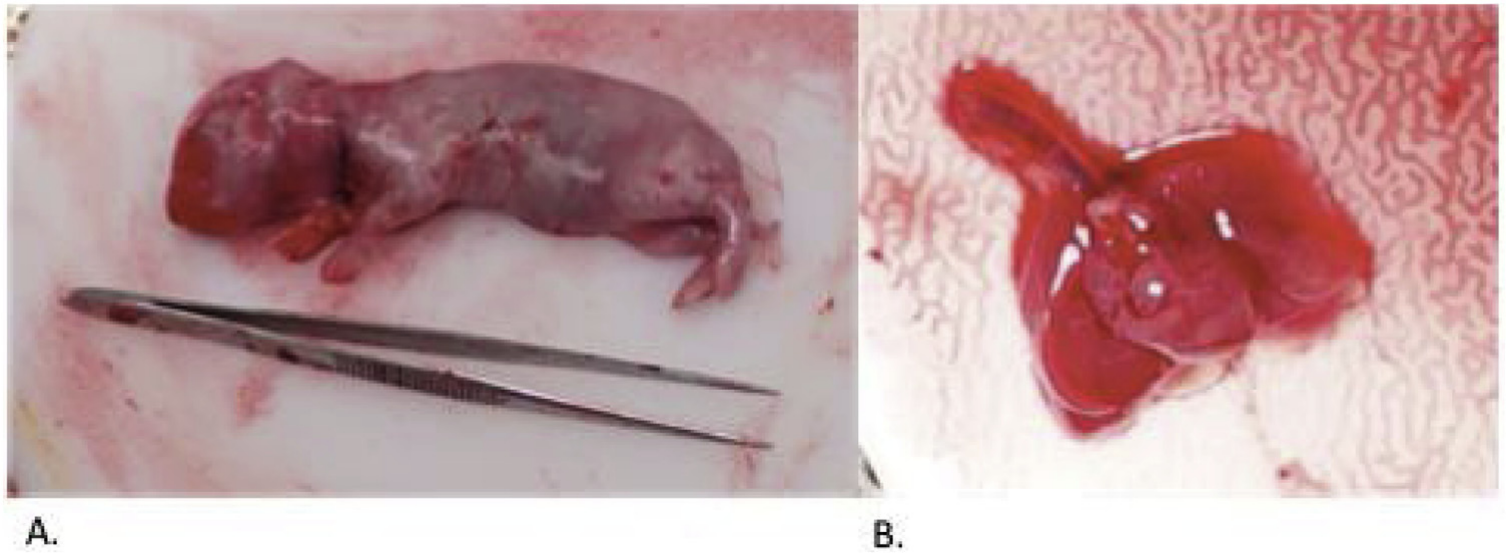
**Autopsy Specimens.** A: CDH+TO fetus as indicated by the sutures noted in the neck and left chest. The fetus has been positioned next to small forceps for size comparison. B: Magnified en bloc section of lungs, heart and trachea. The right and left lungs were further dissected and used in the study whereas the trachea and heart were discarded.

**Fig. 6:**
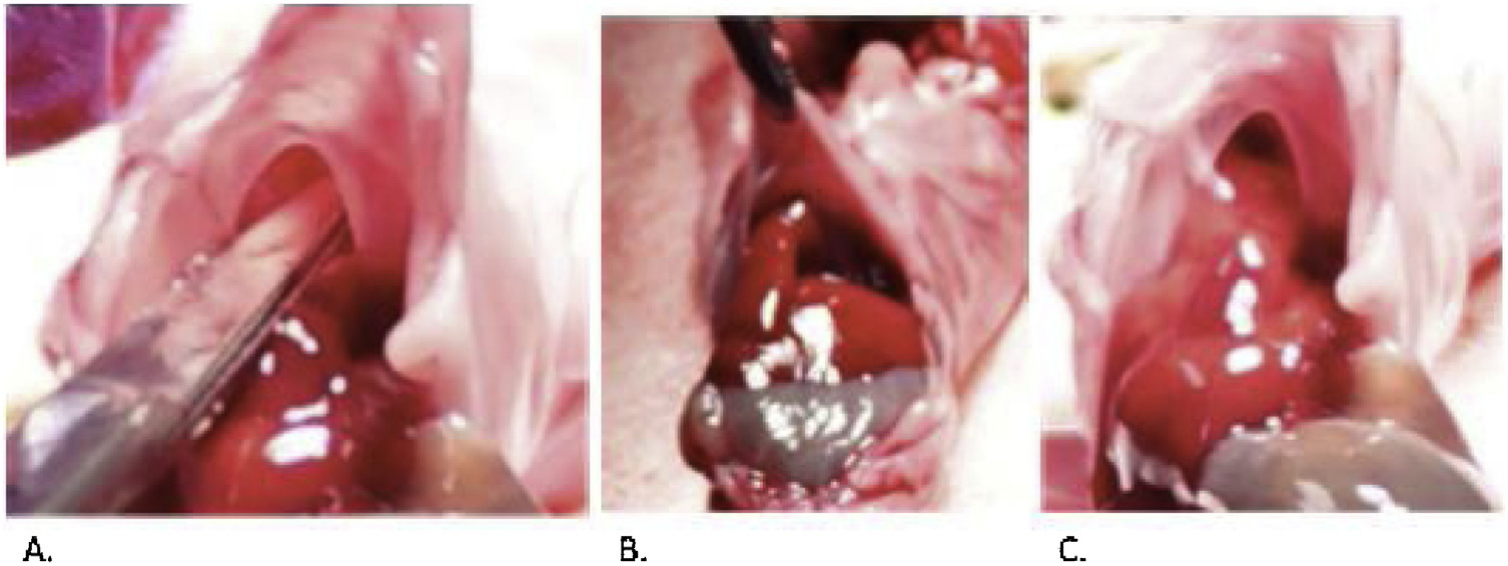
**Autopsy Confirmation of Diaphragmatic Defect.** A: Tip of scissors placed within the diaphragmatic defect to emphasize presence and size of defect. B and C: Intra-abdominal organs herniating into the chest through the diaphragmatic defect.

Fetal weights and lung tissue collection was performed at 31 days gestation. Each doe was euthanized individually in a room away from the remaining does. Each doe was sedated with 10–50 mg/kg Ketamine IM (conc. 100 mg/mL) and 2.5–10 mg/kg Xylazine IM (conc. 100 mg/mL, Bayer Healthcare LLC, Shawnee, KS, USA), and euthanized with 100 mg/kg Pentobarbital Sodium IV (conc. 50 mg/mL, Oak Pharmaceuticals Inc., Lake Forest, IL, USA) administered through the ear vein. A midline laparotomy was made and the doe’s heart was palpated to confirm the absence of cardiac activity. All fetuses were delivered (Fig. 5). The snouts were immediately covered with a saline soaked non-woven sponge and each fetus was decapitated to ensure no fetal breathing. All fetuses were weighed. A sternotomy and midline laparotomy were performed and the diaphragm was assessed for the presence or absence of diaphragmatic defect (Fig. 6). The neck was also incised in CDH + TO fetuses and the trachea was assessed for presence of ligation. One control unoperated fetus was chosen from each doe containing other surgical specimens and was selected based on its weight being the average of the litter. Total lung weight and right lung weight were measured for each fetus and left lung weight was calculated by subtraction (Fig. 5). Upper lung lobes were collected and stored in 4% paraformaldehyde (Electron Microscopy Sciences, Hatfield, PA, USA) and lower lung lobes were flash frozen in liquid nitrogen and stored in −80 °C.

## Method validation

This surgical technique of CDH creation resulted in lung hypoplasia which was reversed by tracheal occlusion [16]. This was recently validated in our research on the effects of tracheal occlusion on Wnt signaling in a rabbit model of CDH [16].
